# Characterization of Volatile Compounds of Eleven *Achillea* Species from Turkey and Biological Activities of Essential Oil and Methanol Extract of *A. hamzaoglui* Arabacı & Budak

**DOI:** 10.3390/molecules200611432

**Published:** 2015-06-22

**Authors:** Fatma Pinar Turkmenoglu, Osman Tuncay Agar, Galip Akaydin, Mutlu Hayran, Betul Demirci

**Affiliations:** 1 Department of Pharmaceutical Botany, Faculty of Pharmacy, Hacettepe University, Ankara 06100, Turkey; E-Mail: tagar@hacettepe.edu.tr; 2 Department of Biology, Faculty of Education, Hacettepe University, Ankara 06800, Turkey; E-Mail: agalip@hacettepe.edu.tr; 3 Department of Preventive Oncology, Cancer Institute, Hacettepe University, Ankara 06100, Turkey; E-Mail: mhayran@hacettepe.edu.tr; 4 Department of Pharmacognosy, Faculty of Pharmacy, Anadolu University, Eskisehir 26470, Turkey; E-Mail: bdemirci@anadolu.edu.tr

**Keywords:** *Achillea*, *Achillea hamzaoglui*, *Asteraceae*, essential oil, antioxidant, antimicrobial, GC-MS, principal components analysis (PCA), hierarchical cluster analysis (HCA)

## Abstract

According to distribution of genus *Achillea*, two main centers of diversity occur in S.E. Europe and S.W. Asia. Diversified essential oil compositions from Balkan Peninsula have been numerously reported. However, report on essential oils of *Achillea* species growing in Turkey, which is one of the main centers of diversity, is very limited. This paper represents the chemical compositions of the essential oils obtained by hydrodistillation from the aerial parts of eleven *Achillea* species, identified simultaneously by gas chromatography and gas chromatography-mass spectrometry. The main components were found to be 1,8-cineole, *p*-cymene, viridiflorol, nonacosane, α-bisabolol, caryophyllene oxide, α-bisabolon oxide A, β-eudesmol, 15-hexadecanolide and camphor. The chemical principal component analysis based on thirty compounds identified three species groups and a subgroup, where each group constituted a chemotype. This is the first report on the chemical composition of *A. hamzaoglui* essential oil; as well as the antioxidant and antimicrobial evaluation of its essential oil and methanolic extract.

## 1. Introduction

The genus *Achillea* L. belongs to Asteraceae (Compositae), which is the largest family of vascular plants and distributed throughout the world. *Achillea* is represented by more than 140 perennial herbaceous species worldwide, and is widespread in Europe and temperate areas of Asia and in North America [1]. Forty-seven species grow in Turkey, 24 of which are endemic (51%). Small capitula forming flat clusters at the top of the stem and hairy aromatic leaves are characteristic for the genus [2,3,4].

*Achillea* species, commonly known as “yarrow”, have been used in folk medicine for thousands of years due to numerous medicinal properties. The name of *Achillea* originated from the name of “Achilles” from Greek mythology, as he used yarrow to treat his bleeding ankle and wounds. Today, several therapeutic applications, such as anti-inflammatory, wound healing, spasmolytic and choleretic uses, are approved by scientific experimental results [1,5,6]. In Turkey, various species of the genus are used in wound healing; abdominal pain; stomachache; symptomatic relief of colds, ulcer, and diarrhea; as diuretic; emmenagog; appetizer; carminative; and insecticidal agent [7,8,9,10,11]. Similar ethnomedicinal uses and some veterinary use of some species were also reported for many other regions in the world [1,5,12,13]. Besides medical applications, plants are used as spices and additives in food products, while essential oil and extracts of some species are used for preparation of digestive teas and cosmetic products. Additionally, some species are cultivated and used in gardening or as cutflowers [5,13].

The genus *Achillea* is rich in terpenoids and flavonoids, which are possible bioactive compounds. Monoterpenes were reported to be the major constituents of essential oil of the genus although high levels of sesquiterpenes were quantified [1,12,14]. Among the monoterpenes 1,8-cineole, found in almost every essential oil, was reported to be the most frequently identified component. Furthermore, it was also reported to be the major compound in about one third of yarrow essential oils. Compounds having bornane skeleton such as camphor and borneol were reported to be the second and third most frequently charactarized components of *Achillea* oil and they were described several times as major compounds. Beside α- and β-pinenes, monoterpenes having *p*-menthane, thujone and pinane skeletons, as mentioned, as the most frequent components have been reported. Sesquiterpenes, both hydrocarbones and oxygenated ones, have also been reported in a considerable number of species. Beside chamazulene, β-caryophyllene and its oxides, α-bisabolol and oxides, cubebene, germacrene, eudesmol, and farnesene were characterized in yarrow oil [5,15,16].

According to distribution of genus *Achillea*, two main centers of diversity occur in S.E. Europe and S.W. Asia [16,17]. In accordance with the great diversity in S.E. Europe, diversified essential oil compositions from the Balkan Peninsula have also been reported and revised by Radulovic *et. al.* [16]. However, reports on essential oils of *Achillea* species growing in Turkey, which is located in both S.E. Europe and S.W. Asia, and considered one of the main centers of diversity, are very limited. This is the first report on essential oil composition of *A. hamzaoglui* Arabacı & Budak*,* which is the most recently identified *Achillea* species in Turkey [4]. Antioxidant and antimicrobial activities of essential oil and methanolic extract of *A. hamzaoglui* were also evaluated. In this paper, we have also investigated essential oil composition of *A. biebersteinii* Afan., *A. coarctata* Poir., *A. kotschyi* Boiss. subsp. *kotschyi*, *A. lycaonica* Boiss. & Heldr., *A. millefolium* L. subsp. *millefolium*, *A. schischkinii* Sosn., *A. setacea* Waldst. & Kit., *A. sintenisii* Hub.-Mor., *A. vermicularis* Trin. and *A. wilhelmsii* C.Koch. subsp. *wilhelmsii* growing in Turkey, in addition to that of *A. hamzaoglui*, of which, *A. lycaonica*, *A. schischkinii, A. sintenisii* and *A. hamzaoglui* are endemic species.

## 2. Results and Discussion

### 2.1. Chemical Composition

Eleven *Achillea* essential oils were obtained by hydrodistillation from air dried aerial parts and subsequently analyzed by GC and GC/MS systems. One hundred seventy-six compounds were identified from *Achillea* oils, which constituted 76.1% to 97.8% of the total oil. Identified compounds in *Achillea* oils with their relative percentages are listed in Table 1.

In the oil of the *A. biebersteinii*, 55 components were characterized, representing 95.7% of the total oil. *p*-cymene (18.6%), 1,8-cineole (16.5%), camphor (11.7%), hexadecanoic acid (11.2%) and β-eudesmol (10.1%) were found as main constituents. *A. biebersteinii* is distributed in S. Bulgaria, S.W. and C. Asia [2]. Literature survey revealed that studies on volatile compounds of this plant accumulated on Asian species. Variations in the essential oil composition of this species from Turkey, Iran, Jordan and Azerbaijan have been evaluated and nine groups of chemotypes were proposed by Polatogluet *et al*. [18]: piperitone, camphor, 1,8-cineole; 1,8-cineole, camphor; *cis*-ascaridole, *p*-cymene; camphor, borneol, 1,8-cineole; spathulenol; α-terpineol, spathulenol; *p*-cymene, camphor; mixed group 1-1,8-cineole, camphor, α-fenchene, santolinatriene; and mixed group 2-1,8-cineole, camphor, piperitone, *iso*-ascaridole, *p*-cymene. *A. biebersteinii* oil investigated in this study might be proposed as third mixed group containing *p*-cymene, 1,8-cineole, camphor, as well as high amounts of hexadecanoic acid and β-eudesmol.

A total of 28 compounds were characterized in *A. coarctata* essential oil, representing 97.3% of the total oil with viridiflorol (25.9%), camphor (9.8%), caryophyllene oxide (9.6%), 15-hexadecanolide (9.4%), hexadecanoic acid (8.2%) and β-eudesmol (7.4%). Oxygenated monoterpenes, 1,8-cineole, camphor and borneol were reported in the essential oils of *A. coarctata* obtained from inflorescence and leaves from Greece [19]. A literature survey revealed that there is only one report on the essential oil composition of *A. coarctata* from Turkey indicating 1,8-cineole, camphor and viridiflorol as major constituents [20]. 15-Hexadecanolide and hexadecanoic acid identified in this study, were not previously reported from *A. coarctata*.

**Table 1:** The composition of the essential oils of *Achillea* species. **AB:** *A. biebersteinii*, **AC:** *A. coarctata*, **AH:** *A. hamzaoglui*, **AK:** *A. kotschyi* subsp. *kotschyi*, **AL:** *A. lycaonica*, **AM:** *A. millefolium* subsp. *millefolium*, **ASc:** *A. schischkinii*, **ASe:** *A. setacea*, **ASi:** *A. sintenisii*, **AV:** *A. vermicularis*, and **AW:** *A. wilhelmsii* subsp. wilhelmsii.

No	RRI ^a^	Compound	Content ^b^ %											IM ^c^
			AB	AC	AH	AK	AL	AM	ASc	ASe	ASi	AV	AW	
1	1014	Tricyclene	0.1	-	-	-	-	-	-	-	-	-	0.2	MS
2	1032	α-Pinene	1.2	0.6	0.5	1.0	-	tr	0.2	-	-	-	0.5	RRI, MS
3	1035	α-Thujene	-	-	0.2	-	-	-	-	-	-	-	-	MS
4	1043	Santolinatriene	0.5	-	-	-	-	-	-	-	-	-	-	MS
5	1076	Camphene	1.7	0.7	0.7	0.5	-	0.1	tr	-	-	-	6.1	RRI, MS
6	1118	β-Pinene	0.6	0.6	1.1	1.4	-	0.1	0.3	-	-	-	0.5	RRI, MS
7	1132	Sabinene	-	-	4.0	-	-	0.1	0.3	-	-	-	-	RRI, MS
8	1188	α-Terpinene	-	-	0.5	0.7	-	0.2	0.1	-	-	-	-	RRI, MS
9	1195	Dehydro-1,8-cineole	-	-	0.1	-	-	-	-	-	-	-	-	MS
10	1202	3-Hexanol	-	-	-	-	0.4	-	-	-	-	-	-	MS
11	1203	Limonene	0.2	-	0.1	0.5	-	-	0.1	-	-	-	-	RRI, MS
12	1213	1,8-Cineole	16.5	1.0	24.1	22.5	0.2	4.1	3.4	3.1	0.6	-	4.2	RRI, MS
13	1222	2-Hexanol	-	-	-	-	0.2	-	-	-	-	-	-	MS
14	1255	γ-Terpinene	0.1	-	1.0	1.8	-	0.6	0.4	-	-	-	-	RRI, MS
15	1280	*p*-Cymene	18.6	0.6	0.2	8.4	-	1.4	8.5	-	-	-	1.8	RRI, MS
16	1285	Isoamylisovalerate	-	-	0.3	-	-	-	-	-	-	-	0.3	MS
17	1290	Terpinolene	-	-	0.2	-	-	-	-	-	-	-	-	RRI, MS
18	1294	1,2,4-Trimethyl benzene	0.2	-	-	-	-	-	-	-	-	-	-	MS
19	1299	2-Methylbutyl isovalerate	-	-	-	-	-	-	-	-	-	-	0.2	MS
20	1355	1,2,3-Trimethyl benzene	0.2	-	-	-	-	-	-	-	-	-	-	MS
21	1400	Nonanal	-	-	-	-	-	-	0.1	-	-	-	-	MS
22	1400	Tetradecane	-	-	-	-	-	-	-	-	-	0.1	-	RRI, MS
23	1405	Santolina alcohol	1.5	-	-	-	-	-	-	-	-	-	-	MS
24	1445	Filifolone	0.3	-	-	-	-	-	0.1	-	-	-	-	MS
25	1474	*trans*-Sabinene hydrate	-	-	2.1	0.5	-	0.5	0.5	-	-	-	0.3	MS
26	1495	Bicycloelemene	-	-	-	-	-	-	0.5	-	-	-	-	MS
27	1497	α-Copaene	-	-	0.4	-	-	-	0.4	-	-	-	-	MS
28	1499	α-Campholene aldehyde	0.3	-	-	-	-	-	-	-	-	-	-	RRI, MS
29	1506	Decanal	-	-	-	-	-	-	0.1	-	-	-	-	RRI, MS
30	1522	Chrysanthenone	1.3	-	-	-	-	-	-	-	-	-	-	MS
31	1532	Camphor	11.7	9.8	6.7	2.8	1.7	4.0	1.5	4.1	0.5	6.7	41.3	RRI, MS
32	1540	Modhephene	-	-	-	0.4	-	-	-	-	-	-	-	MS
33	1547	Dihydroachillene	0.3	-	-	-	-	-	-	-	-	-	0.2	MS
34	1553	Linalool	2.1	-	12.2	-	-	0.6	0.5	-	-	-	-	RRI, MS
35	1556	*cis*-Sabinene hydrate	-	-	1.6	-	0.4	0.4	0.3	-	-	-	0.3	MS
36	1571	*trans-p*-Menth-2-en-1-ol	0.4	-	0.2	-	-	0.3	0.1	-	-	-	-	MS
37	1582	*cis*-Chrysanthenyl acetate	-	-	-	-	2.9	0.5	-	-	0.3	-	-	MS
38	1586	Pinocarvone	0.5	-	0.2	-	-	-	0.2	-	-	-	0.9	RRI, MS
39	1591	Bornyl acetate	0.2	-	-	1.3	-	-	-	-	-	5.1	1.5	RRI, MS
40	1599	Chrysanthenyl propionate	-	-	-	-	-	-	-	-	-	0.1	-	MS
41	1600	Hexadecane	-	-	-	-	0.3	-	-	-	-	0.1	-	RRI, MS
42	1600	β-Elemene	-	-	-	-	-	-	0.1	-	-	-	-	RRI, MS
43	1611	Terpinen-4-ol	0.3	0.7	3.4	1.2	0.6	2.4	-	tr	-	-	0.6	RRI, MS
44	1612	β-Caryophyllene	-	-	1.5	-	-	-	2.8	-	-	-	-	RRI, MS
45	1617	Lavandulyl acetate	-	-	-	-	-	1.1	-	0.9	-	3.3	-	RRI, MS
46	1638	*cis-p*-Menth-2-en-1-ol	0.3	-	0.2	-	-	0.2	0.1	-	-	-	-	MS
47	1648	Myrtenal	0.4	-	0.2	-	-	-	-	0.9	-	-	0.6	MS
48	1651	Sabinaketone	-	-	-	-	0.4	-	-	-	-	-	-	MS
49	1655	Isobornyl propionate	-	-	-	-	-	-	-	-	-	-	0.2	MS
50	1656	Chrysanthenylisobutyrate	-	-	-	-	-	-	-	-	-	0.1	-	MS
51	1661	Alloaromadendrene	-	0.7	0.2	-	-	-	0.4	-	-	-	0.3	MS
52	1664	Nonanol	-	-	-	-	-	-	0.1	-	-	-	-	MS
53	1670	*trans*-Pinocarveol	0.4	-	-	-	0.5	-	0.2	-	-	-	0.6	RRI, MS
54	1678	*cis-p*-Mentha-2,8-dien-1-ol	-	-	-	-	-	-	-	-	-	-	-	MS
55	1682	δ-Terpineol	0.3	-	0.7	0.4	-	0.2	0.1	-	-	-	0.1	MS
56	1683	*trans*-Verbenol	-	-	1.3	-	0.5	-	-	-	-	-	0.6	MS
57	1686	Lavandulol	-	-	-	-	-	0.3	-	-	-	-	-	RRI, MS
58	1687	α-Humulene	-	-	-	-	-	-	0.4	-	-	-	-	RRI, MS
59	1689	*trans*-piperitol	0.3	-	-	-	-	-	-	-	-	-	-	MS
60	1688	Selina-4,11-diene (=*4,11-Eudesmadiene*)	-	-	-	-	-	-	0.1	-	-	-	-	MS
61	1695	(*E*)-β-Farnesene	-	-	0.2	-	-	-	-	-	-	-	-	MS
62	1700	Heptadecane	-	-	-	-	-	-	-	-	-	0.2	-	RRI, MS
63	1704	γ-Muurolene	-	-	-	-	-	-	0.1	-	-	-	-	MS
64	1704	Myrtenyl acetate	-	-	-	-	-	-	-	0.4	-	-	-	MS
65	1705	Fragranyl acetate	-	-	-	-	-	1.0	-	-	-	-	-	MS
66	1706	α-Terpineol	0.6	1.2	2.9	1.6	-	2.2	1.5	0.8	-	0.1	0.2	RRI, MS
67	1715	Geranylformate	-	-	-	-	-	-	0.3	-	-	-	-	MS
68	1719	Borneol	0.6	2.1	0.6	1.0	0.5	0.4	0.3	-	1.3	2.5	6.2	RRI, MS
69	1722	Cabreuva oxide-II	-	-	-	-	-	-	0.2	-	-	-	-	MS
70	1726	Germacrene D	0.1	-	6.2	-	-	-	0.2	-	-	0.1	-	MS
71	1733	Neryl acetate	-	-	-	-	-	0.3	-	-	-	0.6	-	RRI, MS
72	1740	α-Muurolene	-	-	-	-	-	-	0.2	-	-	-	-	MS
73	1742	β-Selinene	-	-	-	-	-	-	-	1.0	0.3	-	0.4	MS
74	1743	Chrysanthenyl isovalerate I	-	-	-	-	0.4	-	-	-	-	4.8	-	,MS
75	1748	Piperitone	1.3	-	-	-	-	-	1.4	-	-	-	-	RRI, MS
76	1755	Bicyclogermacrene	-	-	2.8	-	-	-	0.2	-	-	-	-	MS
77	1758	*cis*-Piperitol	0.3	-	-	0.5	-	0.2	0.2	-	-	-	-	MS
78	1760	Chrysanthenylisovalerate II	-	-	-	-	-	-	-	-	-	3.3	-	MS
79	1764	*cis*-Chrysanthenol	-	-	-	-	2.9	1.5	-	-	-	-	-	MS
80	1768	Cabreuva oxide-IV	-	-	-	-	-	-	0.2	-	-	-	-	MS
81	1770	Isobornylisovalerate	-	-	-	-	-	-	-	-	-	-	0.6	MS
82	1773	δ-Cadinene	-	-	0.2	-	-	1.4	0.2	-	-	-	-	MS
83	1776	γ-Cadinene	-	-	-	-	0.5	-	0.1	-	-	0.1	-	MS
84	1783	β-Sesquiphellandrene	-	-	-	-	-	-	-	-	-	0.1	-	MS
85	1786	*ar*-Curcumene	-	-	-	-	-	-	-	0.2	-	1.3	-	MS
86	1786	Aromadendra-1(10),4(15)-diene	-	-	-	-	-	-	0.6	-	-	-	-	MS
87	1800	Octadecane	-	-	-	-	-	-	-	-	-	0.1	-	RRI, MS
88	1802	Cumin aldehyde	0.6	-	-	0.6	-	-	0.2	-	-	-	-	RRI, MS
89	1804	Myrtenol	0.2	-	0.2	-	-	-	-	1.7	-	-	0.4	MS
90	1807	Fragranol	-	-	-	-	-	2.2	-	-	-	-	-	MS
91	1808	Nerol	-	-	-	-	-	0.6	-	-	-	-	-	RRI, MS
92	1823	*p*-Mentha-1(7),5-dien-2-ol	-	-	-	-	-	-	0.1	-	-	-	-	MS
93	1830	Tridecanal	-	-	-	-	0.1	-	0.2	-	-	-	-	MS
94	1849	Calamenene	-	-	-	-	-	0.2	-	-	-	-	-	MS
95	1857	Geraniol	-	-	-	-	-	-	0.2	-	-	-	-	RRI, MS
96	1868	(*E*)-Geranyl acetone	-	-	-	-	0.2	-	0.1	-	-	0.1	-	MS
97	1898	1,11-Oxidocalamenene	-	-	-	-	-	0.5	-	-	-	-	-	MS
98	1900	*epi*-Cubebol	-	-	-	-		-	tr	-	-	-	-	MS
99	1900	Nonadecane	-	-	-	-	-	-	-	-	-	0.1	-	RRI, MS
100	1921	α-Phellandrene epoxide	-	-	-	-	-	-	0.3	-	-	-	-	MS
101	1941	α-Calacorene	-	-	-	-	-	1.1	0.1	-	-	-	-	MS
102	1945	1,5-Epoxy-salvial(4)14-ene	-	-	-	-	3.9	-	-	-	0.4	1.1	-	MS
103	1953	Palustrol	-	0.8	-	-	-	-	-	-	-	-	-	MS
104	1957	Cubebol	-	-	-	-	0.3	-	0.1	-	-	-	-	MS
105	1958	(*E*)-β-Ionone	-	-	-	-	-	-	-	-	-	0.6	-	MS
106	1969	*cis*-Jasmone	0.5	-	-	-	0.3	-	-	-	-	-	-	MS
107	1984	γ-Calacorene	-	-	-	-	-	0.2	-	-	-	-	-	MS
108	2001	Isocaryophyllene oxide	-	-	0.2	-	-	-	0.3	-	-	-	-	MS
109	2008	Caryophyllene oxide	0.4	9.6	3.9	10.1	3.2	7.7	17.5	3.1	7.5	0.7	3.1	RRI, MS
110	2030	Methyl eugenol	-	-	-	-	-	-	-	-	-	0.1	-	RRI, MS
111	2037	Salvial-4(14)-en-1-one	-	-	-	-	1.7	-	0.2	-	0.6	0.4	-	MS
112	2041	Pentadecanal	0.2	-	0.2	-	1.0	0.3	-	0.5	0.7	0.7	0.2	MS
113	2050	(*E*)-Nerolidol	-	-	2.2	1.6	-	1.1	6.2	-	0.4	4.4	-	MS
114	2056	13-Tetradecanolide	0.2	-	-	-	0.7	-	-	0.8	-	-	1.5	MS
115	2057	Ledol	-	1.6	-	-	-	0.5	0.7	-	-	-	-	MS
116	2071	Humulene epoxide-II	-	0.4	-	0.5	0.5	0.8	1.1	-	0.3	-	-	MS
117	2074	Caryophylla-2(12),6(13)-dien-5-one	-	0.8	-	0.9	0.2	-	0.5	-	0.6	-	1.8	MS
118	2080	Junenol (=*Eudesm-4(15)-en-6-ol*)	-	-	-	-	-	-	0.2	-	-	-	-	MS
119	2088	1-*epi*-Cubenol	-	-	-	-	-	4.1	-	-	-	-	-	MS
120	2092	β-Oplopenone	-	-	-	-	0.5	-	-	-	-	-	-	MS
121	2104	Viridiflorol	-	25.9	-	3.9	-	-	0.2	-	-	-	-	MS
122	2130	Salviadienol	-	-	-	-	0.8	-	-	-	-	-	-	MS
123	2131	Hexahydrofarnesyl acetone	0.3	-	-	0.8	3.3	0.2	0.6	0.8	0.7	1.1	0.6	MS
124	2144	Spathulenol	0.5	1.3	2.0	2.6	4.6	2.5	9.1	2.2	2.7	3.3	0.4	MS
125	2156	α-Bisabolol oxide B	-	-	-	-	-	0.7	-	5.7	-	-	-	MS
126	2161	Bisabolol oxide	-	-	-	-	-	0.6	-	1.1	-	-	-	RRI, MS
127	2161	Muurola-4,10(14)-dien-1-ol	-	-	-	-	-	6.8	0.2	-	-	-	-	MS
128	2179	1-Tetradecanol	-	-	-	-	0.4	-	0.1	-	-	0.7	-	MS
129	2185	γ-Eudesmol	0.1	-	-	-	-	-	-	-	-	-	-	MS
130	2186	Eugenol	0.1	-	-	-	-	-	-	-	-	-	-	RRI, MS
131	2187	T-Cadinol	-	-	0.4	1.0	1.1	-	1.2	-	-	-	0.6	MS
132	2191	Zingiberenol	-	-	-	-	-	-	-	0.4	-	-	-	MS
133	2198	Thymol	0.5	-	-	0.6	-	-	1.2	-	-	-	2.1	RRI, MS
134	2200	α-Bisabolon oxide A	-	-	-	-	-	-	-	27.0	-	-	-	MS
135	2204	Eremoligenol	-	-	-	-	-	-	0.8	-	-	-	-	MS
136	2209	T-Muurolol	-	-	0.3	-	-	-	-	-	-	0.2	-	MS
137	2210	Copaborneol	-	-	-	-	0.8	-	-	-	-	-	-	MS
138	2211	Clovenol	-	0.6	-	-	-	-	0.3	-	-	-	-	MS
139	2214	*ar*-Turmerol	-	-	-	-	0.5	-	-	0.6	-	0.8	-	MS
140	2221	Isocarvacrol	0.4	-	-	-	-	-	-	-	-	-	-	MS
141	2232	α-Bisabolol	-	-	-	-	-	11.7	-	4.8	-	0.5	-	RRI, MS
142	2239	Carvacrol	1.4	-	-	-	-	-	0.6	-	-	-	0.3	RRI, MS
143	2241	Heptadecanal	-	-	-	-	0.9	-	-	-	-	-	-	MS
144	2247	*trans*-α-Bergamotol	-	-	-	-	-	-	0.4	-	-	-	-	MS
145	2255	α-Cadinol	-	-	-	0.3	-	-	-	-	-	-	-	RRI, MS
146	2256	Cadalene	-	-	-	-	-	1.4	-	-	-	-	-	MS
147	2257	β-Eudesmol	10.1	7.4	1.9	4.9	-	-	3.4	-	26.4	4.4	-	RRI, MS
148	2260	15-Hexadecanolide	0.6	9.4	-	-	2.2	-	-	-	-	19.6	0.9	MS
149	2264	Intermedeol	-	-	-	0.7	-	-	-	-	-	-	-	MS
150	2279	Pentadecanol	-	-	-	-	-	-	-	0.7	-	-	-	MS
151	2300	Tricosane	0.9	0.8	-	0.7	1.3	0.4	0.7	1.2	2.4	1.4	0.4	RRI, MS
152	2316	Caryophylla-2(12),6(13)-dien-5β-ol (=*Caryophylladienol I*)	-	0.9	1.0	0.6	0.1	0.8	1.5	-	1.6	-	2.3	MS
153	2324	Caryophylla-2(12),6(13)-dien-5α-ol (=*Caryophylladienol II*)	-	4.3	2.9	2.4	2.7	2.6	4.9	1.2	4.8	-	6.4	MS
154	2325	Bisabolone	-	-	-	-	-	-	-	0.7	-	-	-	RRI, MS
155	2369	Eudesma-4(15),7-dien-4β-ol	-	-	-	-	0.6	0.6	0.9	-	0.6	1.0	-	MS
156	2384	Hexadecanol	-	-	-	-	-	-	-	0.5	-	0.6	-	MS
157	2389	Caryophylla-2(12),6-dien-5α-ol (=*Caryophyllenol I*)	-	2.9	-	1.1	1.1	-	1.2	-	0.5	-	0.7	MS
158	2392	Caryophylla-2(12),6-dien-5β-ol (=*Caryophyllenol II*)	-	2.9	1.0	1.7	1.5	1.2	3.8	-	2.4	-	2.7	MS
159	2400	Tetracosane	-	-	-	-	-	-	-	-	0.5	0.8	-	RRI, MS
160	2411	4-Isopropyl-6-methyl-1-tetralone	-	-	-	-	-	0.6	-	-	-	-	-	MS
161	2430	Chamazulene	-	-	-	-	-	0.4	-	-	-	-	-	MS
162	2438	α-Bisabolol oxide A	-	-	-	-	-	-	-	0.7	-	-	-	MS
163	2475	1-Heptadecanol	-	-	-	-	-	-	-	-	-	1.0	-	MS
164	2500	Pentacosane	1.4	0.7	-	1.2	6.1	1.5	0.2	2.2	3.7	4.4	1.3	RRI, MS
165	2607	1-Octadecanol	-	0.8	-	-	1.0	-	-	-	2.2	2.0	0.6	MS
166	2607	Octadecanol	-	-	-	0.5	-	-	-	-	-	-	-	MS
167	2622	Phytol	-	-	-	-	1.0	0.4	-	-	-	0.9	-	MS
168	2670	Tetradecanoic acid	0.8	-	-	0.7	-	-	-	-	1.4	-	-	RRI, MS
169	2676	(*Z*)-Octadec-9-en-18-olide	0.3	-	-	-	1.3	-	-	-	-	-	-	MS
170	2679	Manool	-	-	-	-	-	-	-	-	-	0.3	-	MS
171	2700	Heptacosane	1.0	-	-	1.7	9.2	1.8	0.7	1.6	4.1	6.3	1.1	RRI, MS
172	2795	Eicosanol	-	-	-	-	-	-	-	-	1.7	-	-	MS
173	2800	Octacosane	-	-	-	-	-	-	-	-	1.3	-	-	RRI, MS
174	2822	Pentadecanoic acid	0.4	-	-	-	-	-	-	-	-	-	-	RRI, MS
175	2900	Nonacosane	0.2	-	-	tr	10.6	2.6	0.4	1.3	2.6	4.1	1.1	RRI, MS
176	2931	Hexadecanoic acid	11.2	8.2	-	7.7	-	4.6	1.9	16.4	22.7	-	0.6	RRI, MS
		**Monoterpene Hydrocarbones**	39.5	3.5	32.7	36.8	0.2	6.6	13.3	3.1	0.6	-	13.3	
		**Oxygenated Monoterpenes**	26.6	13.8	32.5	10.5	11	18.9	9.8	8.8	2.1	26.8	56.4	
		**Sesquiterpene Hydrocarbones**	0.1	0.7	11.5	0.4	0.5	4.3	6.4	1.2	0.3	1.6	0.7	
		**Oxygenated Sesquiterpenes**	11.1	59.4	15.8	32.3	24.1	42.2	55.4	47.5	48.8	16.8	18	
		**Diterpenes**	-	-	-	-	1.0	0.4		-	-	1.2	-	
		**Fatty acid + esters**	12.4	8.2	-	8.4	-	4.6	1.9	16.4	24.1	-	0.6	
		**Others**	6.0	11.7	0.5	4.9	39.3	7.8	3.1	9.6	19.9	43.9	8.8	
		*Identified components*	**55**	**28**	**45**	**41**	**48**	**57**	**82**	**31**	**30**	**46**	**46**	
		*Total*	**95.7**	**97.3**	**93.0**	**93.3**	**76.1**	**84.8**	**89.9**	**86.6**	**95.8**	**90.3**	**97.8**	

^a^ Relative retention indices calculated against *n*-alkanes; ^b^ % calculated from TIC data. ^c^ IM: Identification method based on the relative retention indices (RRI) of authentic compounds on the HP Innowax column; MS, identified on the basis of computer matching of the mass spectra with those of the Wiley and MassFinder libraries and comparison with literature data; tr: <0.1%.

The main components of *A. kotschyi* subsp. *kotschyi* oil were determined as 1,8-cineole (22.5%), caryophyllene oxide (10.1%), *p*-cymene (8.4%) and hexadecanoic acid (7.7%). Forty-one components were identified representing 93.3% of the total *A. kotschyi* subsp. *kotschyi* essential oil. Although *A. kotschyi* subsp. *kotschyi* is distributed in Turkey, Bulgaria and Lebanon [2], the literature for the chemical constituents of its essential oil is poor. There is only one report on the essential oil composition of *A. kotschyi* subsp. *kotschyi*. According to this report, 22 components were characterized, of which 1,8-cineole and caryophyllene oxide were reported as major components [21]. Other major volatile compounds, such as *p*-cymene and hexadecanoic acid, were identified and reported in the present study for the first time.

Nonacosane (10.6%), heptacosane (9.2%) and pentacosane (6.1%) were main constituents of the oil of *A. lycaonica*. Forty-eight components were characterized representing 76.1% of the total oil. Previous data on *A. lycaonica* essential oil is very limited. Thirteen compounds were identified in *A. lycaonica* essential oil and l-camphor, artemisia alcohol and camphor, were reported as major components of the oil [22]. In another study, trans-sabinene hydrate, terpinen-4-ol and caryophyllene oxide were reported to be main constituent of *A. lycaonica* essential oil [23].

A total of 57 compounds were characterized in *A. millefolium* subsp. *millefolium* essential oil, representing 84.8% of the total oil. This oil which was characterized by a high content of α-bisabolol (11.7%), caryophyllene oxide (7.7%) and muurola-4,10(14)-dien-1-ol (6.8%) was dominated by oxygenated sesquiterpenes. *A. millefolium* subsp. *millefolium*, which is an officinal species, is registered in many pharmacopoeias and monograph books. Therefore numerous papers describing the chemical composition of essential oil of *A. millefolium* subsp. *millefolium* from different regions of the world have been written. The major components were reported in different locations as follows: d-cadinene, limonene oxide, alloaromadendrene, caryophyllene oxide, trans-caryophyllene [24] and 1,8-cineol, camphor, α-terpineol, β-pinene, borneol [25] in Turkey; α-asarone, β-bisabolene, α-pinene [26] and 1,8-cineole, sabinene, α-terpineol, terpinen-4-ol, γ-eudesmol, 6*S*,7*R*-bisabolone [27] in Italy; *trans*-thujone, trans-chrysanthenyl acetate, β-pinene in Portuguese [26], 1,8-cineole, bornyl acetate, γ-terpinene, terpinolene in Macedonia [28]; 1,8-cineole, camphor in Serbia [29], β-pinene, sabinene, 1,8-cineole, β-caryophyllene, (*E*)-nerolidol, guaiol, chamazulene in Estonia [30]; borneol, camphor; chamazulene, β-pinene; *trans*-nerolidol, β-pinene and β-pinene, 1,8-cineole in Lithuania [31]; 1,8-cineole [32] and 1,8-cineole, germacrene D in Iran [33], camphor in Kazakhstan [34]; camphor, 1,8-cineole, germacrene D, cis-chrysanthenyl acetate in India [35]; and β-pinene, 1,8-cineole, borneol, β-caryophyllene in Mt. Himalaya (India) [36].

The main components of *A. schischkinii* oil were determined as caryophyllene oxide (17.5%), spathulenol (9.1%), *p*-cymene (8.5%) and (*E*)-Nerolidol (6.2%). Eighty-two components were identified, representing 89.9% of the total *A. schischkinii* essential oil. Donmez *et al*., previously reported 31 components, in the essential of *A. schischkinii* [37]. In this study, 1,8-cineole and camphor were found to be major compounds while caryophyllene oxide, spathulenol and nerolidol (correct isomer was not identified) were reported in small quantities (0.2%–1.1%). On the other hand, *p*-cymene was not identified in *A. schischkinii*. According to Iscan *et al*. [38], 44 components were characterized, and of which 1,8-cineole was identified as the main constituent while caryophyllene oxide, spathulenol and *p*-cymene were reported in lesser quantity (less than 0.1%). (*E*)-Nerolidol was not identified and reported in mentioned study.

In the oil of *A. setacea*, a sum of 31 components were characterized representing 86.6% of the total oil, with α-bisabolon oxide A (27.0%) and hexadecanoic acid (16.4%) as the main constituents. 1,8-cineole and sabinene were previously reported as major components in *A. setacea* [39]. Noteworthy, α-bisabolon oxide A and hexadecanoic acid were not reported either in major or minor quantities previously. 

β-eudesmol (26.4%), hexadecanoic acid (22.7%) and caryophyllene oxide (7.5%) were main constituents of the oil of *A. sintenisii*. Thirty components were characterized representing 95.8% of the total oil. A literature survey revealed that there is only one report on the essential oil composition of *A. sintenisii* [40]. According to this study, camphor, 1,8-cineole, β-pinene, borneol and piperitone were reported as main constituents. Minor quantities of caryophyllene oxide were also reported in this study. Our results showed that β-eudesmol and hexadecanoic acid were reported for the first time in *A. sintenisii* essential oil.

A total of 46 compounds were characterized in *A. vermicularis* essential oil, representing 90.3% of the total oil with 15-hexadecanolide (19.6%), camphor (6.7%), heptacosane (6.3%) and bornyl acetate (5.1%). 1,8-cineole and camphor were previously reported as main components in *A. vermicularis* from Iran and Turkey [18,41], whereas, in our study, 1,8-cineole was not detected and identified in the essential oil of these species.

In the oil of the *A. wilhelmsii* subsp. *Wilhelmsii*, 46 components were characterized representing 97.8% of the total oil. Camphor (41.3%), caryophylladienol II (6.4%), borneol (6.2%) and camphene (6.1%) were found as the other main constituents. There are several different reports on the essential oil composition of *A. wilhelmsii* subsp. *wilhelmsii* from Egypt and different locations of Iran. According to the report on A. *wilhelmsii* growing in Egypt, the major component of essential oil was 1,8-cineole, while camphor, 1,8-cineole, α-pinene; camphor, 1,8 cineole, α-pinene; caryophyllene oxide, camphor, borneol; carvacrol, linalool, 1,8-cineole; camphor, borneol and 1,8-cineole were the major identified compounds in this plant essential oil collected from Iran [42,43,44,45,46]. On the other hand, camphor was previously reported to be main component in the oil of *Achillea wilhelmsii* from Turkey [22].

In addition to these findings, we have investigated essential oil composition of *A. hamzaoglui*, and evaluated antioxidant and antimicrobial activities of both essential oil and methanol extract of the plant. This is the first report on chemical composition of *A. hamzaoglui* essential oil and data is given in Table 1. Forty-five components were identified representing 93% of the total *A. hamzaoglui* essential oil. The main components were determined as 1,8-cineole (24.1%), linalool (12.2%) camphor (6.7%) and germacrene D (6.2%).

### 2.2. Principal Components Analysis (PCA) and Hierarchical Cluster Analysis (HCA)

**Figure 1:**
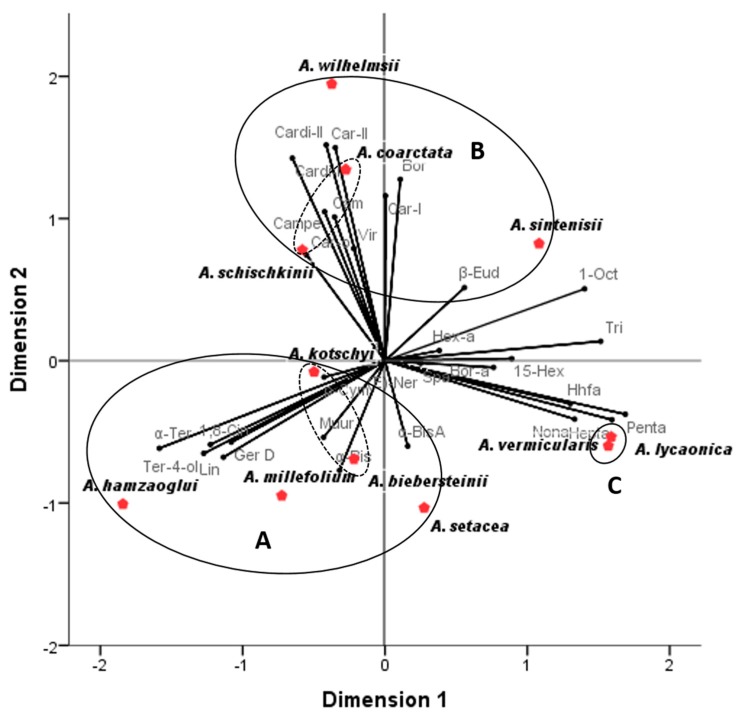
PCA biplot of major volatile compounds of eleven *Achillea* species according to the clusters (**A–C**) they belong to. Axes refer to scores for the samples and loadings from the volatile constituents represented as vectors from the origin. 1-Oct: 1-Octadecanol; 1,8-Cin: 1,8-Cineole; 15-Hex: 15-Hexadecanolide; α-BisA: α-Bisabolon oxide A; α-Bis: α-Bisabolol; α-Ter: α-Terpineol; β-Eud: β-Eudesmol; *p*-Cym*: p*-Cymene; Bor: Borneol; Bor-a: Bornyl acetate; Campe: Camphene, Cam: Camphor; Car-I: Caryophylla-2(12),6-dien-5α-ol; Car-II: Caryophylla-2(12),6-dien-5β-ol; Cardi-I: Caryophylla-2(12),6(13)-dien-5β-ol; Cardi-II: Caryophylla-2(12),6(13)-dien-5α-ol; Car-o: Caryophyllene oxide; (*E*)-Ner: (*E*)-Nerolidol; Ger D: Germacrene D; Hepta: Heptacosane; Hex-a: Hexadecanoicacid; Hhfa: Hexahydrofarnesyl acetone; Lin: Linalool; Muur: Muurola-4,10(14)-dien-1-ol; Nona: Nonacosane; Penta: Pentacosane; Spa: Spathulenol; Ter-4-ol: Terpinen-4-ol; Tri: Tricosane; and Vir: Viridiflorol.

Thirty compounds were included in the PCA and HCA analysis with average concentrations ranging between 0.6%–8.25%. The PCA analysis showed that the first two factorial plans retained, 24.8% and 17.1% of total variance in the data, respectively (Figure 1).

**Figure 2:**
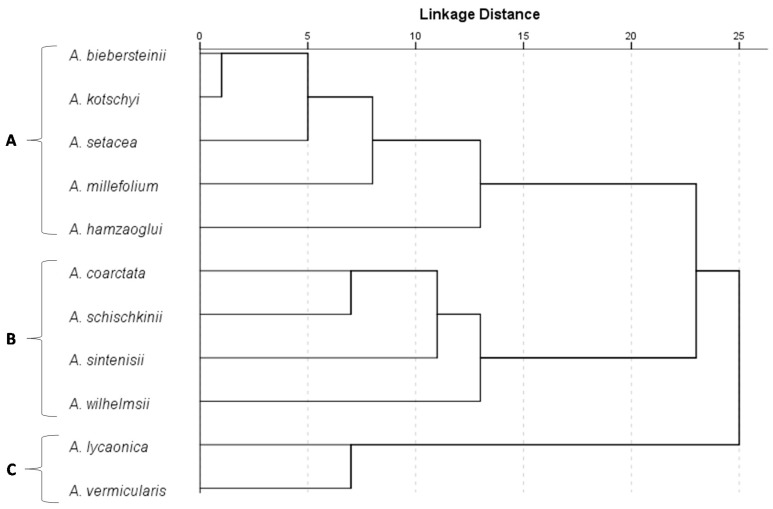
Dendrogram obtained by HCA based on the Euclidean distance between groups of the essential oils of eleven *Achillea* species growing in Turkey. *A. setacea* essential oil was characterized by the highest percentages of two main components in group A: α-bisabolon oxide A and hexadecanoic acid (27% and 16.4%, respectively) and a moderate content of α-bisabolol (4.8%). The essential oil of *A. millefolium* subsp. *millefolium* essential oil was characterized by the highest percentages of α-bisabolol and muurola-4,10(14)-dien-1-ol (11.7% and 6.8%, respectively) as well as its caryophyllene oxide and hexadecanoic acid content (7.7% and 4.6%, respectively) while *A. hamzaoglui* essential oil was characterized by the highest levels of 1,8-cineol, linalool, germacrene D (24.1%, 12.2% and 6.2%, respectively) in group A.

HCA based on the Euclidean distance between groups indicated a solution with three clusters. The number of clusters (A, B and C, Figure 2) were determined by using the rescaled distances in the dendrogram, using a cut-off point where the distances among clusters that are combined increase substantially, indicating the treshold where combining more clusters would increase within-group variability excessively in terms of volatile composition. These clusters formed separate groups in the PCA biplot (Figure 1). The horizontal axis correlated positively with cluster C. The vertical axis correlated positively with cluster B and negatively with cluster A. Essential oils with an average of more than 5% concentration were 1,8-cineol (14.1%), hexadecanoicacid (8.0%), camphor (5.9%), *p*-cymene (5.7%), α-bisabolon oxide A (5.4%), and caryophyllene oxide (5.0%) for cluster A; camphor (13.3%), caryophyllene oxide (9.4%), β-Eudesmol (9.3%), hexadecanoicacid (8.4%), viridiflorol (6.5%), and caryophylla-2(12),6(13)-dien-5α-ol (5.1%) for cluster B; and 15-hexadecanolide (10.9%), heptacosane (7.8%), nonacosane (7.4%), and pentacosane (5.3%) for cluster C.

Group A species comprised *A. biebersteinii*, *A. kotschyi* subsp. *kotschyi*, *A. setacea*, *A. millefolium* subsp. *millefolium* and *A. hamzaoglui*. According to PCA analysis, *A. biebersteinii* and *A. kotschyi* subsp. *kotschyi*, forms a subgroup in group A, because of their high contents of 1,8-cineole (16.5% and 22.5%, respectively), *p*-cymene (18.6% and 8.4%, respectively), hexadecanoic acid (11.2% and 7.7%, respectively) and relatively moderate contents of β-eudesmol (10.1% and 4.9%, respectively). One of the main variations between the subgroup and three other species was due to bornyl acetate content of *A. biebersteinii* and *A. kotschyi* subsp. *kotschyi* (0.2% and 1.3%, respectively) against 0% for the three other species. The *A. biebersteinii* essential oil was richer in camphor (11.7%), whereas *A. kotschyi* subsp. *kotschyi* oil was richer in caryophyllene oxide (10.1%) and viridiflorol (3.9%).

Group B was represented by *A. coarctata*, *A. schischkinii*, *A. sintenisii* and *A. wilhelmsii* subsp. *wilhelmsii*. *A. coarctata* essential oil was characterized by the highest percentages of viridiflorol and 15-hexadecanolide (25.9% and 9.4%, respectively) in group B. It also contains relatively higher amounts of camphor, caryophyllene oxide, hexadecanoic acid and β-eudesmol (9.8%, 9.6%, 8.2% and 7.4%, respectively). *A. schischkinii* essential oil was the richest oil in terms of caryophyllene oxide, spathulenol and (*E*)-nerolidol contents (17.5%, 9.2% and 6.2%, respectively), whereas *A. sintenisii* essential oil was the richest one in terms of β-eudesmol and hexadecanoic acid (26.4% and 22.7%, respectively). *A. sintenisii* essential oil also contains relatively moderate percentage of caryophyllene oxide (7.5%). In Group B, *A. wilhelmsii* subsp. *wilhelmsii* was characterized by the highest percentages of camphor, caryophylla-2(12),6(13)-dien-5α-ol, borneol and camphene (41.3%, 6.4%, 6.2% and 6.1%, respectively).

Group C, represented by *A. lycaonica* and *A. vermicularis*. *A. lycaonica* was characterized by higher percentages of nonacosane, heptacosane and pentacosane (10.6%, 9.2% and 6.1%, respectively) while *A. vermicularis* essential oil was characterized by the highest percentage of 15-hexadecanolide (19.6%). *A.vermicularis* was also richer in terms of camphor, bornyl acetate and (*E*)-nerolidol content (6.7%, 5.1% and 4.4%, respectively).

In the genus *Achilea*, composition of the essential oil is highly variable due to some biotic and abiotic factors, such as ontogenic and morphogenic differentiations, environmental factors and applied method of oil extraction [47]. Previous studies on the oil composition of *Achillea* species revealed that 1,8-cineole was the most abundant compound, ranging from trace levels to 47.7% in essential oils of Balkan *Achillea*, while camphor and borneol were the second and third repeatedly detected compounds, respectively. Moreover, caryophyllene oxide and β-caryophyllene were reported to be frequently identified sesquiterpenoids [5,15,16]. According to our results, all *Achillea* species, except *A. vermicularis*, investigated in this study contain 1,8-cineole from 0.2% to 24.1%. Contrary to Balkan *Achillea*, camphor, which was detected in all species between 0.5% and 41.3%, was found to be the most abundant compound in this study. In addition to camphor, caryophyllene oxide and spathulenol were detected in all species. 1,8-cineole, borneol, tricosane and pentacosane were the second most detected compounds (Table 1). Oxygenated sesquiterpenes such as β-eudesmol, viridiflorol, spathulenol, nerolidol, caryophyllene oxide, caryophylladienol II, α-bisabolon oxide, α-bisabolol and muurola-4,10(14)-dien-1-ol were found to be major components of investigated species. Among sesquiterpenes, chamazulene was regarded as characteristics of the members of Millefolium group (Syn: sect. *Achillea*) by Radulovic *et al*. [16]. However, according to some other researchers, this is not accepted as a universal phenomenon in the group because some species belonging Millefolium group was reported most of the cases as chamazulene free, moreover, some species outside the group contain chamazulene [15,47]. In paralel to this agreement, in the present study, among five species belonging Millefolium group, *A. biebersteinii*, *A. coarctata*, *A. kotschyi* subsp. *kotschyi*, *A. millefolium* subsp. *millefolium* and *A. setacea*, chamazulene was detected only in *A. millefolium* subsp. *millefolium* (0.4%). Futhermore, chamazulene was reported as one of the major components of essential oil of *A. millefolium* subsp. *millefolium* [30,31], while in many cases, the species was chamazulene free [24,25,32]. Cubebene, which was reported previously from *Achillea* species [15], was not identified in this study. Besides sesquiterpenes, in this study, some fatty acid derived compounds such as 15-hexadecanolide, hexadecanoic acid, nonacosane, heptacosane and pentacosane were found to be as major compounds in nine species (in minor quantities in *A. schischkinii* and *A. millefolium* subsp. *millefolium*) (Table 1). Noteworthy, major compounds of *A. lycaonica* investigated in this study were consists of fatty acid derived compounds. 

### 2.3. Antioxidant and Antimicrobial Activity of Essential Oil and Methanol Extract of A. hamzaoglui

Free radicals, which are continuously produced in human body as normal products of cellular metabolism, are essential for several physiological processes in low concentrations. However, in higher amounts they can react with membrane lipids, nucleic acids, proteins, enzymes and other small molecules and cause human diseases, including cancer, diabetes, atherosclerosis, failures in immunity and endocrine functions [48]. Antioxidants act as safeguard against the accumulation of free radicals and their elimination from the system. In addition, due to their important role in living systems, antioxidants have been widely used in cosmetics and foods [49]. In the present study, free radical scavenging activity of characterized essential oil (AH-EO) and methanolic extract (AH-ME) of *A. hamzaoglui* was evaluated by DPPH method in comparison with that of a synthetic antioxidant, tert-butylhydoxy-toluene (BHT), at different concentrations [50]. Radical scavenger activity was expressed as the amount of antioxidants necessary to decrease the initial DPPH absorbance by 50% (median effective concentration value, EC_50_) (Table 2). Beside DPPH method, total antioxidant capacity (TAC) of methanol extracts and essential oil was also evaluated. The TAC assay, which is a single assay sufficient for reliable determination of antioxidant potential of a complex sample, is based on the reduction of copper (II) to copper (I) by antioxidants [51]. TAC values were shown as mM reducing equivalents to uric acid (UAE) and as μM copper reducing equivalents (CRE) in Table 2.

**Table 2:** Antioxidant activity of essential oil and methanol extract of *A. hamzaoglui* *.

Sample	DPPH Scavenging Assay	Total Antioxidant Capacity	
	EC_50_ (μg/mL)	UAE ^1^ (mM)	CRE ^2^ (μM)
AH-EO	-	0.082 ± 0.003	179.50 ± 6.57
AH-ME	32.09 ± 1.98	2.038 ± 0.011	4461.18 ± 24.08
BHT	29.83 ± 1.23	-	-

*: Results are represented as means ± standard deviation (*n* = 3); ^1^: Uric acid equivalent, ^2^: Copper reducing equivalent.

As far as antioxidative potency of the samples is concerned, AH-ME was more effective in both DPPH and TAC assays. AH-ME reduced the stable free radical DPPH with a very low EC_50_ value (32.09 ± 1.98 μg/mL), which was very similarto that of reference compound BHT (EC_50_ = 29.83 ± 1.23 μg/mL). EC_50_ value of AH-EO could not be calculated because of lower values of inhibition than 50%. Total antioxidant capacity of AH-ME and AH-EO was measured as 2.038 ± 0.011 UAE and 0.082 ± 0.003 URE, respectively, showing that the AH-ME is 25-fold stronger than AH-EO.

*In vitro* antimicrobial activity of AH-EO and AH-ME against common Gram-positive and Gram-negative bacteria and standard *Candida* strains associated with human infections was assessed by using broth microdilution method [52,53]. Minimum inhibitory concentrations (MIC) of the test samples and standards are summarized in Table 3. According to the microdilution assay, AH-ME showed relatively weak antimicrobial effects against all tested bacteria (MIC; 0.625 mg/mL) except *Pseudomonas aeruginosa* (MIC; 0.15625 mg/mL). In comparison to Chloramphenicol, Clarithromycin and Tetracycline, AH-EO showed moderate to weak inhibitory effects (MIC; 0.15625–0.625 mg/mL) against the tested bacteria, except *Staphylococcus aureus* (MIC; 0.07812 mg/mL), which was strongly inhibited by AH-EO. Both AH-ME and AH-EO also showed moderate inhibitory effects on the *Candida* species (MIC; 0.15625–0.3125 mg/mL).

Previous investigations of *Achillea* species essential oils demonstrated mostly weak or moderate antimicrobial activity. As there is no data available about the activity of essential oil of *A. hamzaoglui* in the literature, antimicrobial activity can only be directly compared with chemically similar oils, composing at least the same major compounds. However, apart from chemical variations of essential oils, differences in methods applied and diversity of microorganisms used, make results incomparable. In light of these facts, antimicrobial activity of essential oils of some *Achillea* species containing 1,8-cineol as major component were given. Tzakou *et al*., reported that the best inhibitory effect of essential oils of the inflorescences and leaves of *A. coarctata*, which were characterized by the abundance of oxygenated monoterpenes 1,8-cineole (26.9% and 29.1%, respectively), camphor (22.1%, 9.2%) and borneol (5.0% and 6.8%, respectively), was detected against *Micrococcus flavus*, *Enterococcus faecalis* and *C. albicans* (MIC; 3.25 mg/mL) [19]. According to literature survey, essential oils of *A. setaceae* and *A. teretifolia*, whose major oil was 1,8-cineol (18.5% and 19.9%, respectively), exhibited inhibitory effects on *Clostridium perfringens*, *Acinetobacter lwoffii* and *C. albicans* with a range of minimum inhibitory concentration values extended from 0.28 to 2.25 mg/mL [39]. In another study, water-soluble and water-insoluble fractions of methanol extract and essential oil of *A. millefolium,* which contain 1,8-cineol (24.6%), camphor (16.7%), α-terpineol (10.2%), β-pinene (4.2%), and borneol (4.0%) as principal components, were tested against various microorganism. The essential oil possessed stronger antimicrobial activity than the extracts tested. The oil exhibited moderate activity against *Streptococcus pneumoniae*, *Clostridium perfringens* and *C. albicans*, and weak activity against *Mycobacterium smegmatis*, *Acinetobacter lwoffii* and *C. krusei* [25]. In addition, in contrary to our results, same authors also reported that the oil strongly reduced the DPPH radical (IC_50_ = 1.56 μg/mL) while water-soluble part of methanol extract of the plant showed weaker radical scavenging ability (IC_50_ = 45.60 μg/mL). In another study, it was reported that essential oils of *A. teretifolia* and *A. vermicularis*, which were investigated at various concentrations and incubation time, also showed considerable DPPH scavenging activity [18]. DPPH radical scavenging capacity of the floral infusions of *A. biebersteinii*, *A. coarctata*, *A. kotschyi* subsp. *kotschyi*, *A. schischkinii*, *A. setacea*, and *A. teretifolia*, which are growing in Turkey, were reported to be 33.5%, 23.9%, 27.4%, 33.6%, 27.4% and 28.7%, respectively, while total antioxidant capacity of infusions, based on the reduction of Mo (VI) to Mo (V), were determined to be 8.419, 4.671, 5.599, 8.419, 6.999 and 6.928 mMα-Tocopherol/100 mL, respectively [54]. In another study, ethanol extracts of aerial parts of Iranian *A. millefolium*, *A. vermicularis* and *A. wilhelmsii* exhibited DPPH radical scavening activity with EC_50_ = 49.43, EC_50_ = 85.28 and EC_50_ = 118.90 [55]. There are no previous data on the antioxidant potential of *A. lycaonica* and *A. sintenisii* extracts.

**Table 3:** Antimicrobial activity of essential oil and methanol extract of *A. hamzaoglui.*

Microorganisms	MIC *(μg/mL)					
	AH-EO ^1^	AH-ME ^2^	Chloramphenicol	Clarithromycin	Tetracycline	Ketoconazole
**Bacteria**						
*Escherichia coli* (NRRL B3008)	625	625	2	-	-	-
*Salmonella typhimurium* (ATCC 13311)	312.5	625	4	-	-	-
*Pseudomonas aeruginosa* (ATCC 10145)	156.25	156.25	32	-	-	-
*Staphylococcus aureus* (ATCC BAA-1026)	78.12	625	8	-	-	-
*Propionibacterium acnes* (ATCC 6919)	156.25	625	4	0.25	1	-
*Streptococcus mitis* (NCIMB 13770)	312.5	625	2	0.25	0.5	-
**Fungi**						
*Candida krusei* (NRRLY 7179)	312.5	312.5	-	-	-	0.5
*Candida albicans* (ATCC 24433)	312.5	312.5	-	-	-	0.25
*Candida tropicalis* (ATCC 1369)	312.5	312.5	-	-	-	0.25
*Candida parapsilosis* (ATCC 22019)	312.5	156.25	-	-	-	0.25

* Minimum inhibition concentrations, ^1^ *A. hamzaoglui* essential oil, ^2^ *A. hamzaoglui* methanol extract.

Natural antioxidants are generally more desirable for consumption than synthetic ones because these natural antioxidants avoid the toxicity problems which may arise from the use of synthetic antioxidants, such as butylated hydroxy anisole (BHA) and butylated hydroxy toluene (BHT) [49]. Therefore, in order to find new sources of safe antioxidants of natural origin, plant extracts have been extensively searched for their antioxidant and radical scavenging properties. In the present study, AH-ME exhibited distinctively strong DPPH radical scavenging activity with a EC_50_ = 32.09 ± 1.98 μg/mL as well as high total antioxidant capacity (2.038 ± 0.011 UAE) when compared our results with previous data on *Achillea* species.

## 3. Experimental Section

### 3.1. Plant Material

The plants were collected during the flowering stage from different provinces of inner Anatolia. The collected samples were identified and the voucher specimens were deposited in the Herbarium of Hacettepe University, Faculty of Pharmacy (HUEF) and Herbarium of Hacettepe University Faculty of Education (HEF). Information concerning the plant material is given in Table 4.

**Table 4:** Herbaria records of *Achillea* species.

*Achillea* sp.	Collection Site and Altitude	Collection Period	Specimen Number *
*A. biebersteinii*	A4 Ankara: Beytepe Campus, the road into the forest, 39°52ʹ40ʹʹN, 32°43ʹ49ʹʹE, 992 m	26 June 2014	HUEF 14053
*A. coarctata*	B5 Nevşehir: Nevşehir to Aksaray, 2 km to Camiören village, 38°30ʹ15ʹʹN, 34°23ʹ19ʹʹE, 1220 m	22 July 2013	HEF 15093
*A. hamzaoglui*	B5 Kırşehir: Kırşehir to Mucur, Kervansaray Mountain, junction of Bahçecik, Mehtap Hill, 39°08ʹ22ʹʹN, 34°17ʹ46ʹʹE, 1350 m	1 June 2013	HEF 14970
*A. kotschyi* subsp. *kotschyi*	B6 Yozgat: Akdağmadeni, above Kızılcaova village, Nalbant hill, 39°31ʹ14ʹʹN, 36°01ʹ20ʹʹE, 2000 m	1 July 2014	HUEF 14044
*A. lycaonica*	B6 Sivas: Ulaş, Ziyarettepe, 39°32ʹ44ʹʹN, 37°02ʹ11ʹʹE, 1450 m	24 July 2013	HEF 15095
*A. millefolium* subsp. *millefolium*	B6 Yozgat: Akdağmadeni, Karababa mountain, South of Çerçialan village, 39°32ʹ27ʹʹN, 36°06ʹ60ʹʹE, 1880 m	4 August 2014	HUEF 14047
*A. schischkinii*	B6 Sivas: Şerefiye, Karabayır Passage, 40°09ʹ50ʹʹN, 37°50ʹ53ʹʹE, 1925 m	25 July 2013	HEF 15097
*A. setacea*	B6 Sivas: Zara, Avşar village, near Arapça, 39°58ʹ48ʹʹN, 37°45ʹ09ʹʹE, 1300 m	6 July 2014	HUEF 14046
*A. sintenisii*	B6 Sivas: Ulaş, Ziyarettepe, 39°33ʹ41ʹʹN, 37°00ʹ52ʹʹE, 1430 m	7 July 2014	HUEF 14056
*A. vermicularis*	B9 Van: Van-Bahçesaray yolu, Sisar deresi mevkii, 38°15ʹ51ʹʹN 43°10ʹ23ʹʹE, 2015 m	12 July 2013	HUEF 13013
*A. wilhelmsii* subsp. *wilhelmsii*	C5 Niğde: Ovacık village, Ovacık-Çamardı road, 38°06ʹ07ʹʹN, 34°49ʹ17ʹʹE, 1350 m	9 July 2014	HUEF 14050

* HUEF: Herbarium of Hacettepe University Faculty of Pharmacy, HEF: Herbarium of Hacettepe University Faculty of Education.

### 3.2. Isolation of Essential Oil and Extraction

The air-dried aerial parts of the plant material were hydrodistilled for 3 h using a Clevenger-type apparatus to produce a small amount (<0.01%) of volatiles, which was trapped in *n*-hexane. Air-dried and ground plant material of *A. hamzaoglui* were subjected to hydrodistillation for 3 h using a Clevenger-type apparatus to obtain essential oil in 0.07% yield. All samples were stored at 4 °C in the dark until analyzed. Twenty grams of plant material was also extracted with methanol three times at 40 °C and methanol was evaporated under reduced pressure (yield 11.6% w/w).

### 3.3. Gas Chromatography Analysis

The GC analysis was carried out using an Agilent 6890N GC system. FID detector temperature was 300 °C. To obtain the same elution order with GC/MS, simultaneous auto injection was done on a duplicate of the same column applying the same operational conditions. Relative percentage amounts of the separated compounds were calculated from FID chromatograms.

### 3.4. Gas Chromatography-Mass Spectrometry Analysis

The GC/MS analysis was carried out with an Agilent 5975 GC/MSD system. Innowax FSC column (60 m × 0.25 mm, 0.25 μm film thickness) was used with helium as carrier gas (0.8 mL/min). GC oven temperature was kept at 60 °C for 10 min and programmed to 220 °C at a rate of 4 °C/min, and kept constant at 220 °C for 10 min and then programmed to 240 °C at a rate of 1 °C/min. Split ratio was adjusted at 40:1. The injector temperature was set at 250 °C. Mass spectra were recorded at 70 eV. Mass range was from *m/z* 35 to 450.

### 3.5. Identification of Components

Identification of the oil components was carried out by comparison of their relative retention times with those of authentic samples or by comparison of their relative retention index (RRI) to series of n-alkanes. Computer matching against commercial (Wiley and MassFinder 3) [56,57] and in-house “Başer Library of Essential Oil Constituents” built up by genuine compounds and components of known oils, as well as MS literature data [58] were also used for the identification.

### 3.6. Antioxidant Activity

#### 3.6.1. DPPH Radical Scavenging Assay

The free radical scavenging activity of the fractions was measured *in vitro* by 2,2′-diphenyl-1-picrylhydrazyl (DPPH) assay according to the method described earlier [50]. The stock solution was prepared by dissolving 24 mg DPPH (Sigma-Aldrich, St. Louis, MO, USA) with 100 mL methanol and stored at 20 °C until required. The working solution was obtained by diluting DPPH solution with methanol to attain an absorbance of about 0.98 ± 0.02 at 517 nm using the spectrophotometer. A 3 mL aliquot of this solution was mixed with 100 μL of the sample at various concentrations (1–150 μg/mL). The reaction mixture was shaken well and incubated in the dark for 30 min at room temperature. Then the absorbance was taken at 517 nm. The scavenging activity was estimated based on the percentage of DPPH radical scavenged as the following equation:

DPPH radical scavenging activity (%) = [(A_0_ − A_1)_/A_0_] × 100 (1)

**A**_0_: Absorbance of the control at 30 min (517 nm); **A**_1_: Absorbance of the sample at 30 min (517 nm). BHT (Sigma-Aldrich) was used as a positive control. Tests were carried out in triplicate. Afterwards, a curve of % DPPH scavenging capacity *versus* concentration was plotted and EC_50_ values were calculated. EC_50_ denotes the concentration of sample required to scavenge 50% of DPPH free radicals.

#### 3.6.2. Total Antioxidant Capacity (TAC) Assay

The assay was carried out using commercial TAC assay kit (OxiSelect™ Total Antioxidant Capacity (TAC) Assay Kit, Cell Biolabs, Inc., San Diego, CA, USA). Upon reduction, the copper (I) ion further reacts with a coupling chromogenic reagent that produces a color with a maximum absorbance at 490 nm. The net absorbance values of antioxidants are compared with a known uric acid standard curve. Absorbance values are proportional to the sample’s total reductive capacity. Results are expressed as μM copper reducing equivalents or mM uric acid equivalents. A fresh uric acid standard was prepared by weighing out the uric acid powder for a 10 mg/mL solution in 1 N NaOH. This 10 mg/mL is equivalent to a concentration of 60 mM. The 60 mM uric acid solution was used to prepare a 2 mM solution of uric acid (e.g., add 100 μL of the 60 mM uric acid standard to 2.9 mL of deionized water). Each sample was prepared using the stock solution of 100 mg/mL concentration. An initial reading was taken at 490 nm. Then, 50 μL of the 1× copper ion reagent was added and incubated for 5 min on an orbital shaker. Then, 50 μL of the stop solution was added to terminate the reaction and the plate was read again at 490 nm. All determinations were performed in triplicate and results were averaged.

### 3.7. Antimicrobial Activity

#### 3.7.1. Test Microorganisms

Microorganisms were obtained from ATCC, NRRL or clinical isolates (Eskişehir Osmangazi University, Turkey; Anadolu University, Faculty of Science, Department of Biology, Eskişehir, Turkey) and were stored in 15% glycerol containing micro-test tubes at −86 °C (strain numbers of microorganisms were given in Table 3). All microorganism strains were inoculated on Mueller Hinton Agar (MHA) or Sabouraud Dextrose Agar (SDA) prior the experiments at 37 °C. After sufficient growth, the microorganisms were transferred to Mueller Hinton Broth (MHB) for further incubation at the same conditions for another 24 h [52,53].

#### 3.7.2. Broth Microdilution Assay

Test samples as well as standard antimicrobial controls were first dissolved in DMSO (25%) at an initial concentration. Serial dilution series were prepared in 100 µL Mueller Hinton Broth (MHB) with an equal amount of the test samples. Overnight grown microorganism suspensions at appropriate conditions were first diluted in double strength MHB and standardized turbitometrically to 1 × 5 10^6^–10^8^ CFU/mL (McFarland No: 0.5) under sterile conditions. Then each microorganism suspension was pipetted into each well and incubated at 37 °C for 24 h. Sterile distilled water and medium served as a positive growth control. The first well without turbidity was assigned as the minimum inhibitory concentration (MIC, in µg/mL). To visualize the antimicrobial activity Tetrazolium Violet 1% (w/v, EtOH) (2,5-diphenyl-3-[α-naphthyl] tetrazolium chloride, TTC, Sigma) was also used. Average results of separately performed three experiments were given [52,53].

### 3.8. Statistical Analysis

The essential oils components useful in reflecting chemotaxonomic and biological relationships, compounds detected in oil samples with an average concentration of greater than 0.6% were selected. The components were subjected to a principal component analysis (PCA) and to hierarchical cluster analysis (HCA) using IBM SPSS software version 22.0.

## 4. Conclusions

In this study, we investigated essential oil compositions of *A. hamzaoglui* along with ten *Achillea* species growing in Turkey, which is one of the main diversity centers of the genus *Achillea*. With regard to their essential oil composition, PCA and HCA analysis enabled to identify three groups and a subgroup of *Achillea* species, where each group constituted a chemotype.

There is a widespread agreement that synthetic antioxidants need to be replaced with natural ones because some synthetic antioxidants have shown potential health risks and toxicity. Therefore, in order to find new sources of safe antioxidants of natural origin, a great interest has been given to the antioxidant and radical scavenging properties of plant extracts. In the present study, *A. hamzaoglui* methanol extract exhibited very strong radical scavenging activity and high antioxidant capacity. The findings indicate that methanol extract of *A. hamzaoglui* can be considered a rich natural source of antioxidants that could be used in pharmaceutical preparations, cosmetics and foods. On the other hand, essential oil of the plant exhibited much more prominent antimicrobial activity against tested microorganisms than that of methanol extract. Because of its strong antibacterial activity against *Staphylococcus aureus* (ATCC BAA-1026) and moderate antibacterial activity against *Pseudomonas aeruginosa* (ATTC 10145) and *Propionibacterium acnes* (ATCC 6919), the essential oil could be effectively used for skin infections. This study is the first report on essential oil composition and antioxidant and antimicrobial activities of *A. hamzaoglui,* and calls for further investigations to elucidate its effect on other biological activities.
